# Examination of Local Functional Homogeneity in Autism

**DOI:** 10.1155/2015/174371

**Published:** 2015-06-09

**Authors:** Lili Jiang, Xiao-Hui Hou, Ning Yang, Zhi Yang, Xi-Nian Zuo

**Affiliations:** ^1^Key Laboratory of Behavioral Science, Institute of Psychology, Chinese Academy of Sciences, No. 16 Lincui Road, Chaoyang District, Beijing 100101, China; ^2^Magnetic Resonance Imaging Research Center, Institute of Psychology, Chinese Academy of Sciences, No. 16 Lincui Road, Chaoyang District, Beijing 100101, China; ^3^University of Chinese Academy of Sciences, Shijingshan, Beijing 100049, China; ^4^Laboratory for Functional Connectome and Development, Institute of Psychology, Chinese Academy of Sciences, No. 16 Lincui Road, Chaoyang District, Beijing 100101, China

## Abstract

Increasing neuroimaging evidence suggests that autism patients exhibit abnormal brain structure and function. We used the Autism Brain Imaging Data Exchange (ABIDE) sample to analyze locally focal (~8 mm) functional connectivity of 223 autism patients and 285 normal controls from 15 international sites using a recently developed surface-based approach. We observed enhanced local connectivity in the middle frontal cortex, left precuneus, and right superior temporal sulcus, and reduced local connectivity in the right insular cortex. The local connectivity in the right middle frontal gyrus was positively correlated with the total score of the autism diagnostic observation schedule whereas the local connectivity within the right superior temporal sulcus was positively correlated with total subscores of both the communication and the stereotyped behaviors and restricted interests of the schedule. Finally, significant interactions between age and clinical diagnosis were detected in the left precuneus. These findings replicated previous observations that used a volume-based approach and suggested possible neuropathological impairments of local information processing in the frontal, temporal, parietal, and insular cortices. Novel site-variability analysis demonstrated high reproducibility of our findings across the 15 international sites. The age-disease interaction provides a potential target region for future studies to further elucidate the neurodevelopmental mechanisms of autism.

## 1. Introduction

Autism spectrum disorder (ASD) is an increasingly recognized group of neurodevelopmental disorders with early onset and lifelong persistence. ASD is reported to occur in ~1% of children [1] and is characterized by abnormalities in language, social interaction, and a range of stereotyped and repetitive behaviors.

Neuroimaging studies of ASD have accumulated a wealth of empirical data on the abnormal brain connectomics associated with ASD [2–4]. Reduced long distance but increased local connectivity in ASD has been proposed [5], and a number of FMRI studies have consistently found underconnectivity in ASD [2, 6–8]. However, a significant number of other studies report mixed or increased functional connectivity in ASD [9–11]. Regarding these inconsistencies, Muller and colleagues systematically illustrated that different methodological choices could produce different results in functional connectivity studies [4]. Although methodological choices may affect the statistical results of ASD studies, a consistent and reliable demonstration of brain function using a large sample would be a good step towards elucidating the brain mechanisms of ASD.

Using a local connectivity index of functional homogeneity, ReHo, both increases and decreases of ReHo were observed in resting-state FMRI studies of ASD [12, 13]. ReHo is a promising index of human brain function that has been applied to multiple neuropsychiatric disorders [2, 12–14]. It employs Kendall's coefficient of concordance (KCC) to measure the functional coherence between a given area (e.g., a voxel) and its adjacent areas (e.g., neighboring voxels) [15]. Previous studies on spontaneous brain activity in ASD used volume-based ReHo (3dReHo), ignoring the two-dimensional nature of the laminar cerebral cortex [2, 14]. In volume space, voxel's neighbors may not be close to the voxel across the cortical mantle. Recently, our lab developed a surface-based ReHo method (2dReHo) that demonstrated moderate to high test-retest reliability and correlated with neurobiological information processing hierarchies [16, 17]. The present study began by testing the consistency between the previous 3dReHo and our new 2dReHo results across a large-scale autism sample. Furthermore, as revealed by our recent work [17], 2dReHo is a neurobiologically meaningful metric of the functional organization of the human brain. We thus aimed to examine the effects of ASD on brain functional organization.

Abnormal cortical development and organization in children with ASD has been characterized in terms of brain cortical volume, surface area, and cortical thickness [18, 19]. Previous genetic findings, coupled with brain imaging studies, suggested a potential unifying model of ASD in which higher-order association areas of the brain that normally connect to the frontal lobe are partially disconnected during development [20, 21]. In general, autism is conceived as a heterogeneous childhood neurodevelopmental disorder because of its early onset and lifelong persistence. Using 223 ASD and 285 healthy controls (HC) from the autism brain imaging data exchange (ABIDE) lifetime sample [2, 22], we examined both group differences and diagnosis-age interactions in local functional homogeneity measured by 2dReHo, as well as behavioral correlations in ASD.

## 2. Materials and Methods

### 2.1. Participants and MR Imaging

The ABIDE sample was part of the 1000 Functional Connectomes Project (FCP: http://fcon_1000.projects.nitrc.org/fcpClassic/FcpTable.html) and the International Neuroimaging Data-Sharing Initiative (INDI: http://fcon_1000.projects.nitrc.org/indi/pro/nki.html) and included RFMRI images of 539 ASD (aged 17.01 ± 8.37) and 573 healthy controls (aged 17.08 ± 7.72) from 18 international sites [2]. Detailed phenotypic and scanning information can be found at the ABIDE website: http://fcon_1000.projects.nitrc.org/indi/abide/. The overlap of phenotypic protocol across sites consisted of age at scan, sex, IQ, and diagnostic information. All contributions were based on studies approved by the local institutional review boards, and written informed consent was obtained from the parents of all the early onset patients and corresponding healthy controls, as well as all the adult participants.

Similar to the ABIDE consortium paper [2], 794 subjects from the original participant pool were selected for subsequent imaging analyses. The criteria were (1) individuals without other comorbidities; (2) male subjects, as they represent 90% of the ABIDE sample; (3) sites with fIQ estimated for >75% and subjects with fIQ scores; (4) individuals with fIQ within 2 s.d. of the overall ABIDE sample mean (i.e., 108 ± 15); (5) sites with at least 9 participants per group after the above exclusions.

### 2.2. Image Preprocessing

For each participant, all MRI images were preprocessed with the Connectome Computation System (CCS: http://lfcd.psych.ac.cn/ccs.html) [16]. This system was developed based on FCP scripts for providing a computational platform for multimodal brain connectome analysis by integrating AFNI [23, 24], FSL [25], and FreeSurfer [26] and has been used in our previous studies [16, 17].

All structural MRI images were processed with the CCS structural pipeline. Briefly, the structural images were processed for cortical surface reconstruction [27–31], including (1) noise removal with a spatially adaptive nonlocal means filter [32, 33] operation and correction for intensity inhomogeneity; (2) brain extraction with a hybrid watershed/surface deformation; (3) tissue segmentation of the cerebrospinal fluid (CSF), white matter (WM), and deep gray matter (GM); (4) cutting plane generation to disconnect the two hemispheres and subcortical structures; (5) fixation of the interior holes of the segmentation; (6) a triangular mesh tessellation over the GM-WM boundary and mesh deformation to produce a smooth GM-WM interface (white surface) and GM-CSF interface (pial surface); (7) topological defect correction on the surface; (8) individual surface mesh inflated into a sphere; and (9) estimation of the deformation between the resulting spherical mesh and a common spherical coordinate system that aligned the cortical folding patterns across subjects.

All functional images were preprocessed with the CCS functional pipeline, involving the following steps: (1) eliminating the first 5 EPI volumes from each scan to allow for signal equilibration; (2) despiking time series to detect and reduce outliers (spikes) using an hyperbolic tangent function; (3) slice timing using Fourier interpolation to temporally correct the interleaved slice acquisition; (4) aligning each volume to a “base” image (the mean EPI image) using Fourier interpolation to correct between-head movements; (5) normalizing the 4D global mean intensity to 10,000 to allow intersubject comparisons; (6) regressing out the WM/CSF mean time series and the Friston-24 motion time series to reduce the effects of these confounding factors [16, 34]; (7) filtering the residual time series with a passband filter (0.01–0.1 Hz) to extract low-frequency fluctuations; (8) removing both linear and quadratic trends; and (9) aligning individual motion corrected functional images to the individual anatomical image using a GM-WM boundary-based registration (BBR) algorithm [35]. Individual preprocessed 4D RFMRI time series were projected onto the* fsaverage5* standard cortical surface with 10,242 vertices per hemisphere and gaps of 4 mm on average [36].

### 2.3. Quality Control Procedure

The CCS quality control procedure provides an interactive environment for users (http://lfcd.psych.ac.cn/ccs/QC.html) to examine the quality of (1) brain extraction or skull stripping, (2) brain tissue segmentation, (3) pial and white surface reconstruction, (4) functional-structural image realignment with BBR registration, and (5) head motion during RFMRI, calculated using several quantities: (1) the maximum distance of translational head movement (maxTran), (2) the maximum degree of rotational head movement (maxRot), (3) the mean framewise displacement (meanFD) [37, 38], and (4) the minimal cost of the BBR coregistration (mcBBR). All subjects with bad brain extraction, tissue segmentation, and surface reconstruction were excluded from the subsequent computation. All datasets filled the following criteria: (1) maxTran ≤ 2 mm, (2) maxRot ≤ 2°, (3) meanFD ≤ 0.4 mm, and (4) mcBBR< 0.75. We were left with a total of 508 subjects (223 ASD, 285 HC) from 15 different sites passing the above quality control procedure for the final statistical analysis (see Table 1 for the composition of the final sample).

### 2.4. 2dReHo and Statistics

We applied surface-based 2dReHo to characterize local functional homogeneity in both ASD and HC subjects due to its high test-retest reliability [16] neurobiological significance [17]. Specifically, for a given vertex *v*_0_ on the surface grid* fsaverage5*, we identified its *K* nearest neighbors *v*_*i*=1,2,…,*K*_ and denoted by *v*_*i*_(*t*) their RFMRI time series. The 2dReHo measure of this vertex was computed as Kendall's coefficient of concordance (KCC) using these time series. The mathematical formula is shown as (1), where *R*_*i*=1,…,*n*_ represents the ranks of *v*_*i*_(*t*), *n* is the number of time points, *R*_*i*_ is the mean rank across its neighbors at the *i*th time point, and *R* is the overall mean rank across all neighbors and across all the time points. A vertexwise 2dReHo surface map was produced by repeating this computation procedure for every vertex on the surface of both hemispheres. Both length-one (6 neighbors) and length-two (19 neighbors) 2dReHo maps, denoted by 2dReHo1 and 2dReHo2, respectively, were generated for subsequent analyses:

(1) KCC=∑i=1nRi2−nR−21/12K2n3−n=12∑i=1nR−i2n3−n−3n+1n−1.

A general linear model was constructed by modeling diagnosis (ASD versus HC), site, age, and fIQ as covariates of interests (see details in (2)). Notably, before the group level analysis of 2dReHo, we first removed the effects of meanFD, mcBBR, Jacobian values of white surface, and global mean (gm) 2dReHo (see details in (3)). We have found that this computation is equivalent to directly including all these factors in the final group analysis for the purpose of examining the effects of the variables of interest. Here, the mean FD indicates the mean framewise displacement [37] and mcBBR indicates the warp distortion for BBR-based function-to-structure realignment. The vertexwise significance values for group difference and age by group interactions were corrected for multiple comparisons with a clusterwise method based on random field theory (cluster-defining *P* = 0.01, cluster-level corrected *P* = 0.05):

(2) Y=β1×age+β2×site+β3×fIQ +β4×Group+β5×Group×age+e,

(3) Yadj=Y−βgm×gm+βfd×meanFD     + βbbr×mcBBR+βjac×JAC.

### 2.5. Behavioral Correlations

Autism diagnostic observation schedule (ADOS) [39], autism diagnostic interview (ADI), and the Gotham algorithm of the ADOS (ADOS_GOTHAM) were selected for the final behavioral correlation analyses, as each of these subscale scores represented >40% of the whole patient group. Within each cluster showing a significant group difference in 2dReHo, we calculated the Pearson correlation coefficient between the average 2dReHo values of the cluster and the behavioral measurements. ADOS includes the total score (ADOS_TOTAL), the communication total subscore (ADOS_COMM), the social total subscore (ADOS_SOCIAL), and the stereotyped behaviors and restricted interests total subscore (ADOS_STEREO_BEHAV). ADI includes the reciprocal social interaction subscore (A) total for the autism diagnostic interview-revised (ADI_R_SOCIAL_TOTAL_A), the abnormality in communication subscore (B) total for the autism diagnostic interview-revised (ADI_R_VERBAL_TOTAL_BV), the restricted, repetitive, and stereotyped patterns of behavior subscore (C) total for the autism diagnostic interview-revised (ADI_R_RRB_TOTAL_C), and the abnormality of development evidence at or before 36-month subscore (D) total for the autism diagnostic interview-revised (ADI_R_ONSET_TOTAL_D). ADOS_GOTHAM includes the social affect total subscore for the Gotham algorithm of the ADOS (ADOS_GOTHAM_SOC_AFFECT), restricted and repetitive behaviors total subscore for the Gotham algorithm of the ADOS (ADOS_GOTHAM_RRB), the sum of the social affect total and restricted and repetitive behaviors total (ADOS_GOTHAM_TOTAL), and the individual calibrated severity score for the Gotham algorithm of the ADOS (ADOS_GOTHAM_SEVERITY).

## 3. Results

### 3.1. Sample Composition

**Figure 1:**
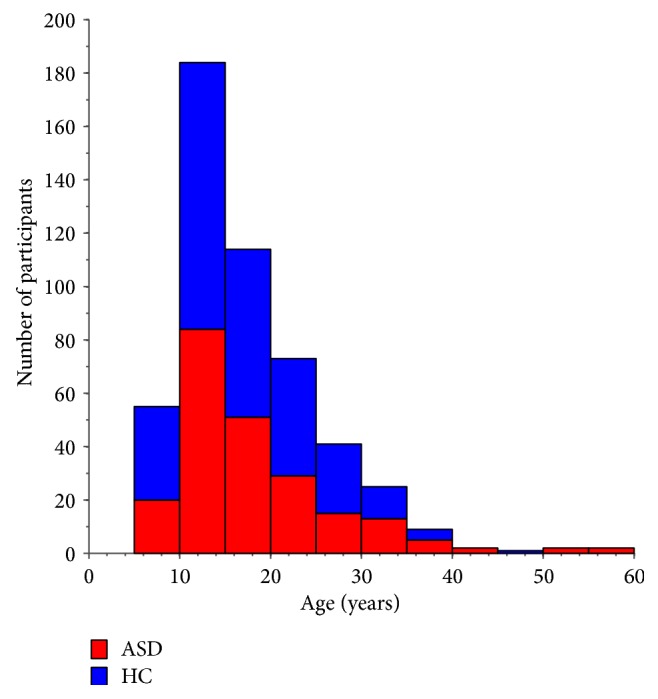
Sample characteristics of the ABIDE sites included in the final analyses.

The final analysis was conducted on 508 participants from 15 sites. The details of the sample composition are presented in Table 1. The number of participants per site ranged from 3 to 35 ASD patients and from 2 to 69 HC subjects. The age distribution is shown in Figure 1. The red and blue bars represent the ASD and HC numbers for a particular age bin, respectively. There is no significant difference in age between the ASD and HC subjects (*t* = 1.40; *P* = 0.16). Results derived with 2dReHo1 and 2dReHo2 were almost identical and we thus presented the findings based upon 2dReHo1, which were detailed in below.

### 3.2. Group Differences in 2dReHo

**Figure 2:**
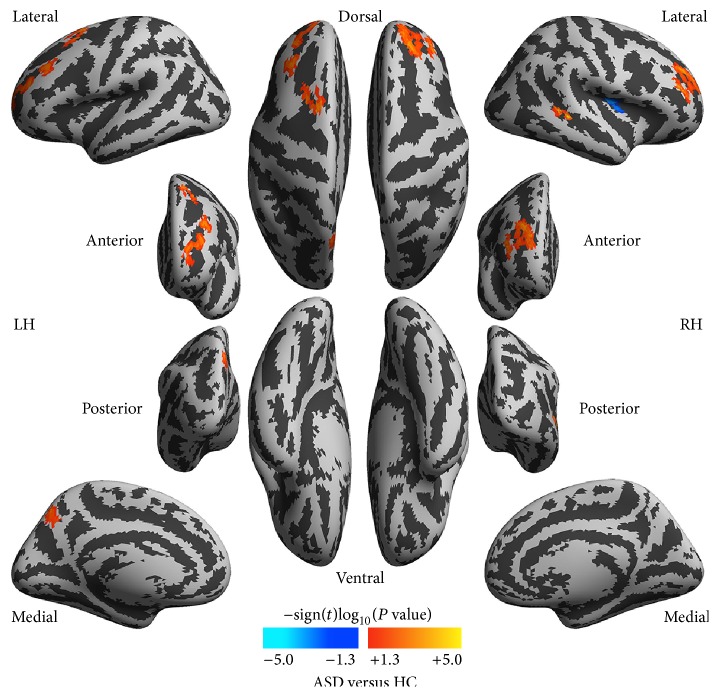
Group differences in local functional homogeneity between autism spectrum disorder (ASD) patients and healthy controls (HC). The vertexwise significance of group comparisons is measured with signed log_10_ transformed *P* values and is rendered onto the cortical surfaces of the left hemisphere (LH) and right hemisphere (RH). These inflated surfaces are defined by FreeSurfer as the* fsaverage5* surface model and visualized from six different (lateral, medial, posterior, anterior, dorsal, and ventral) views. Light gray colors indicate a cortical gyrus whereas dark gray colors show a cortical sulcus.

There was no difference in the global 2dReHo between ASD and HC subjects (*t* = −0.33, *P* = 0.75).  Figure 2 depicts the brain areas that significantly differed between ASD and HC subjects. The anatomical labels and locations are summarized in Table 2. The middle frontal cortex, the left precuneus gyrus, and right superior temporal sulcus exhibited increased local functional homogeneity (warm colors) in ASD compared with HC. The right insular cortex had decreased local functional homogeneity (cool colors) in ASD compared to HC subjects.

### 3.3. Age by Diagnosis Interactions

**Figure 3:**
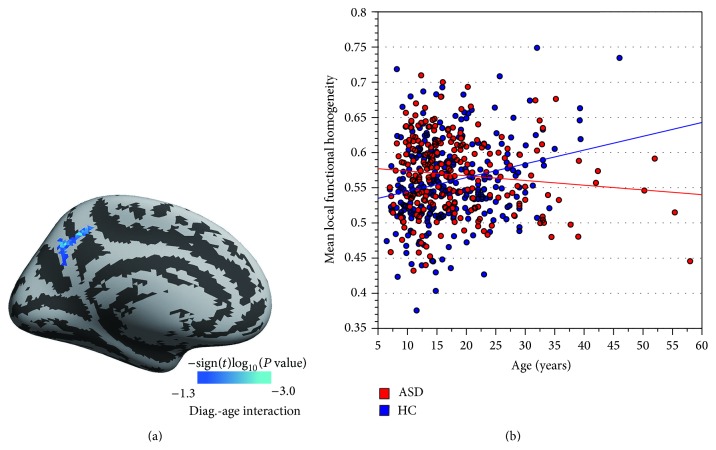
The vertexwise significance (the signed log_10_ transformed *P* values) of interactions between clinical diagnosis (diag.: ASD versus HC) and age is visualized on the left medial cortical surface (a). The details of the diag.-age interaction are further plotted as scatters in (b), where each dot represents the individual mean local functional homogeneity in the left precuneus cluster indicated in (a).

The age-diagnosis interaction was examined for differences in correlations between age and local functional homogeneity (i.e., the developmental effect) between ASD and HC subjects. Significant group by age interaction effects were detected in the left precuneus gyrus (Figure 3(a)), where ASD patients demonstrated lower 2dReHo than HC subjects (Figure 2). To look into the details of 2dReHo changes across this large age span, we also visualized the age dependence of 2dReHo as scatter plots for ASD and HC subjects (Figure 3(b)). Clearly, the scatter plot shows that ASD subjects had increased 2dReHo with age, whereas HC subjects had decreased 2dReHo, the opposite pattern, with age.

### 3.4. Site Effects and Reproducibility

**Figure 4:**
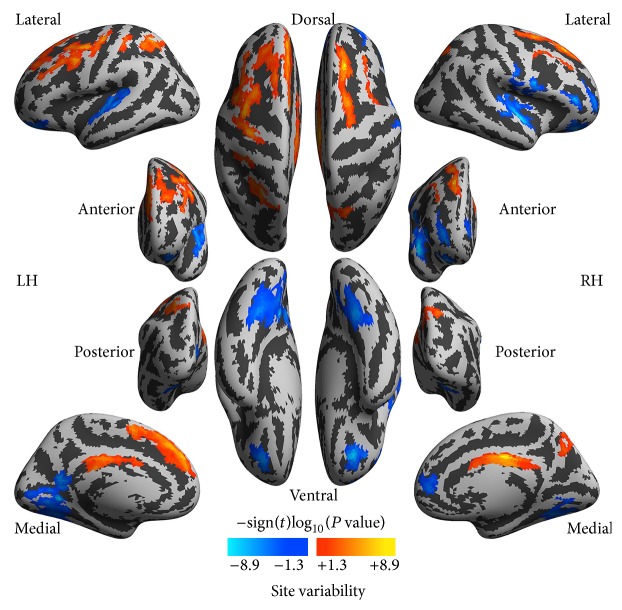
Site variability in local functional homogeneity. The vertexwise significance of site variability is measured with signed log_10_ transformed *P* values and rendered onto the cortical surfaces of the left hemisphere (LH) and right hemisphere (RH). These inflated surfaces are defined by FreeSurfer as the* fsaverage5* surface model and visualized in six different (lateral, medial, posterior, anterior, dorsal, and ventral) views. Light gray colors indicate a cortical gyrus whereas dark gray colors indicate a cortical sulcus.

Because this was a multisite study, we included site as one covariate of interest. Site effects revealed by the statistical model are illustrated in Figure 4. Multiple regions showed significant site effects, implying a remarkable variability of 2dReHo across the 15 international sites. Specifically, the left pre/postcentral gyrus, the middle/inferior frontal gyrus, the left medial prefrontal cortex, the left superior parietal gyrus, and cingulate gyrus exhibited significant site variability in 2dReHo. In contrast, the superior temporal gyrus, the right postcentral gyrus, the inferior part of the precentral sulcus, the inferior frontal sulcus, the right insular cortex, the right precuneus, the left calcarine sulcus, the occipital-temporal gyrus, and right medial prefrontal cortex exhibited an inverse pattern of site variability.

**Figure 5:**
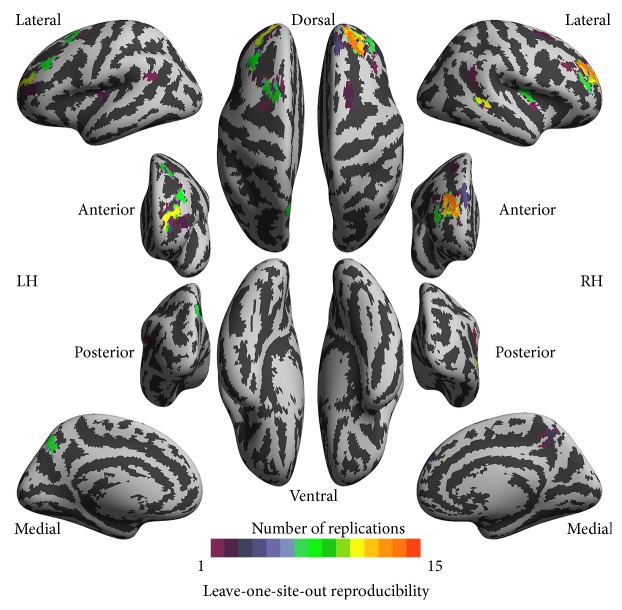
Leave-one-site-out reproducibility of group differences in local functional homogeneity between autism spectrum disorders (ASD) patients and healthy controls (HC). The vertexwise numbers of replications of group comparisons are rendered onto the cortical surfaces of the left hemisphere (LH) and right hemisphere (RH). These inflated surfaces are defined by FreeSurfer as the* fsaverage5* surface model and visualized in six different (lateral, medial, posterior, anterior, dorsal, and ventral) views. Light gray colors indicate a cortical gyrus whereas dark gray colors show a cortical sulcus.

To further investigate the impacts of site variability on the findings presented, we applied a leave-one-site-out validation (LOSOV) approach. Specifically, we left one of the 15 sites out from the ASD-HC comparisons and repeated the group level analyses using the datasets from the other 14 sites. The site reproducibility of the current findings was measured as the number of replications of the voxelwise ASD-HC significance maps.  Figure 5 illustrates the reproducibility of the findings, which were highly replicated for the middle frontal cortex, the right superior temporal sulcus, and left precuneus.

### 3.5. Behavioral Correlations

The mean 2dReHo values within the right middle frontal gyrus were positively correlated with the ADOS_TOTAL scores (*r* = 0.1728; *P* = 0.0171), whereas the mean 2dReHo values within the right superior temporal sulcus were positively correlated with both the ADOS_COMM (*r* = 0.1542; *P* = 0.0428) and ADOS_STEREO_BEHAV (*r* = 0.1620; *P* = 0.0477) scores.

## 4. Discussion

Based upon an aggregated large sample with a wide age range, the ABIDE consortium paper [2] examined different aspects of the functional architecture of autism brains using various derivatives but did not explore age or site effects. The current study quantitatively examined the local functional homogeneity of ASD and age/site effects. Using the ABIDE resting-state FMRI samples of 223 ASD patients and 285 normal controls from 15 different sites, we observed increased local functional homogeneity measured by 2dReHo in the middle frontal cortex, the left precuneus, and the right superior temporal sulcus and decreased 2dReHo in the right insular cortex. Within the left precuneus, ASD patients exhibited decreased 2dReHo with age, but normal controls showed increased 2dReHo with age. Notably, 2dReHo demonstrated a widely distributed spatial pattern of variability across sites. Across-site reproducibility of the observation was thus conducted to show the dependency of our findings, which revealed a novel contribution to assessment to both the variability and reproducibility of the findings across sites in a multicenter design. These findings indicate alterations in the complexity of functional information processing across the associative cortex and further correlate with multiple behavioral outcomes in ASD. The age-disease interaction offers a target region, the left precuneus, for future developmental studies on ASD.

Consistent with the volume-based 3dReHo method used by Di Martino et al. [2], the middle frontal cortex exhibited increased ReHo in ASD patients. The reduction of ReHo in the right insular cortex in ASD is consistent with a previous study of adults with autism [13]. A study on children with autism [12] reported that the right temporal region exhibited greater ReHo in autism compared with typical developing controls. These consistencies and replications validate our computation and analyses and suggest a possible neuropathology in the frontal, temporal, precuneus (parietal), and insular cortices. A widely distributed spatial pattern of local functional organization across the association cortex is disrupted in ASD. There were also some inconsistencies in the group differences in local functional connectivity between ASD patients and healthy controls across the previous studies. The reason may lie in the heterogeneity of the disorder and the different age distribution of the participants, as well as different methodological choices [4]. More importantly, if ASD was indeed characterized by an atypical neurodevelopmental pattern, even a moderate difference in the age range could have a substantial influence on the results [40]. Beyond these inconsistencies, the implication of the spatial patterns of local functional organization will be discussed in the following sections.

We observed increased local functional homogeneity in ASD patients compared to normal controls in the middle frontal cortex, part of the prefrontal cortex. In addition to the above evidence for frontal alterations in ASD with 2dReHo, both the fractional amplitude of low-frequency fluctuations and the degree centrality of human brain function exhibited impaired patterns in the frontal cortex in ASD [2]. During brain development, in ASD, higher-order association areas that normally connect to the frontal lobe are partially disconnected [20], and local connectivity is strengthened while long-distance connectivity is impaired in the frontal cortex [5]. Structural abnormalities, such as a thicker left frontal cortex, have also been reported [19]. Based upon our recent study on the neurobiological significance of ReHo in [17], increased ReHo in the middle frontal cortex may indicate reduced functional segregation or a reduced complexity of local information processing. The frontal cortex is responsible for executive function, which may involve coordination of multiple human brain networks [17]. ASD may involve disrupted functional segregation in this area, which may induce abnormal behaviors in patients.

The superior temporal sulcus showed increased ReHo in ASD compared to normal controls. Similar abnormalities in the superior temporal sulcus have been characterized using cerebral blood flow (CBF), brain morphology, and functional metrics in previous studies. In greater detail, metabolism was reduced, as indicated by decreased rCBF, in autistic patients at the superior temporal regions [41]. A decreased concentration of grey matter in the superior temporal sulcus was observed in autistic children [42]. Directly related to the current report, Shukla and colleagues observed that the right temporal regions exhibited greater ReHo in autism compared with typically developing controls [12]. Using the ABIDE datasets, Anderson and colleagues recently reported the best accuracy of whole brain classification between ASD and HC using intrinsic functional connectivity of Wernicke's area, which is located in the temporal lobe [22]. Increases of 2dReHo in the superior temporal sulcus implied decreases of local functional separation or differentiation [17] and a decreased complexity of information processing [17, 43]. Taking this explanation a step further because this area is a part of the language network [44] and ASD patients exhibit abnormal functional architecture of the superior temporal sulcus, these results may explain impaired language function and social interactions in ASD.

The right insula was the only region with decreased ReHo in ASD patients. Abnormality of the insula is one of the most consistent findings of fMRI studies on ASD. In addition to abnormal ReHo in the insular areas of ASD [13], alterations of VMHC (voxel-mirrored homotopic connectivity) and DC in the insular were observed in ASD [2]. The classification accuracy between ASD and HC using functional connectivity was best in the insular cortex using the same ABIDE data as the current study [22]. Meanwhile, decreased left posterior insular activity during auditory language in autism was reported previously [45]. A longitudinal study on children aged 5–11 years showed decreases in left posterior insular activity with age [46]. Given the neurobiological significance of ReHo [17], decreased ReHo in the insular cortex may indicate decreased functional integration. Considering the roles of the insular region in emotion and decision-making [47] and as a hub in supporting the brain connectome [48, 49], alterations of local functional homogeneity in the insular area may correlate with impaired emotion processing and other high-level cognitive processings in autism.

ASD patients and normal controls displayed different trends of age dependencies. This finding may help to explain the neurodevelopmental mechanisms of ASD. Other studies reported abnormalities in the left precuneus in ASD compared with normal controls. Functional alterations of VMHC [22] and a thicker cortex in the left precuneus gyrus [19] were found in two separate studies. Mak-Fan and colleagues confirmed a similar age by group interaction pattern of overall brain volume, overall grey matter volume, overall surface area, and mean thickness of the brain in ASD and HC [19]. Another study also explored the age by group interaction in ASD focusing on brain morphology [18]. Importantly, the current study found consistent group differences and age by group interactions in the left precuneus gyrus. The left precuneus may therefore be a target region for future developmental studies of ASD.

The increases of local functional homogeneity in the right middle frontal gyrus and right superior temporal sulcus in ASD are associated with the severity of ASD symptoms. Considering both the high test-retest reliability [16] and the emerging neurobiological significance of 2dReHo, we propose that 2dReHo may serve as a neuroimaging marker for the diagnosis, treatment, and prevention of ASD in the future [50]. As illustrated in the group difference subsection of the Discussion, the frontal cortex and superior temporal sulcus are two major areas of autism studies for both brain structure and function [20, 51]. The current findings were also mostly reproducible across different sites for these two regions (Figure 5). The correlations of average ReHo with ADOS symptoms indicate that these regions may be targets for further explorations of the neuropathology of ASD [51].

### 4.1. Limitations and Future Directions

The ABIDE consortium paper [2] reported less commonly explored regions such as the thalamus. In the current study, the subcortical regions and cerebellum were excluded due to the limitation of the surface-based ReHo method. These regions may be a direction of future studies by developing surface-based ReHo approaches for these noncerebral structures. The second limitation of ReHo is its nature of local short-range connectivity and being not suitable for characterizing global long-range connectivity. A sample limitation is that the sample was composed of mainly males with ASD (>90%). Therefore, sex differences may be crucial in ASD progression during brain development, and future studies with a comparable number of male and female ASD patients could improve our understanding of the neuropathology of the disorder. Thirdly, the subgroup of ASD generally included autism, Asperger's syndrome, or PDD-NOS. There is an ongoing debate about clinical standards for the classification of the four subgroups. However, enough samples for each subgroup would give more insight into the classification in terms of neuroimaging-based data mining approaches (see a pilot demonstration in early onset schizophrenia in [52]). Finally, all age-related findings observed in the present study are derived from a cross-sectional dataset and should be interpreted with caution that these age-related changes can be interpreted as developmental effects only with a longitudinal sample in future.

## 5. Conclusions

In the current study, using the ABIDE sample with 223 ASD and 285 healthy controls with wide age span, we observed increased local functional homogeneity ReHo in the middle frontal sulcus and gyrus, the left precuneus gyrus, and right superior temporal sulcus, together with decreased local functional homogeneity ReHo in the right insular. Significant group by age interactions in the left precuneus gyrus were also found, and the group difference (increased 2dReHo in ASD) decreased with age. At the same time, the average 2dReHo values within the right middle frontal gyrus were significantly positively correlated with ADOS_TOTAL scores, and the average 2dReHo values within the right superior temporal sulcus were significantly positively correlated with ADOS_COMM and ADOS_STEREO_BEHAV scores. All of these findings, especially the consistent group differences, the interaction effects in the precuneus gyrus, and the behavioral correlations, contribute to our understanding of the neurodevelopmental pathological mechanisms of ASD.

## Figures and Tables

**Table 1:** ABIDE sample composition in the current study.

Site name	ASD number	HC number	Age (years)
Caltech	4	6	29.5 ± 11.8
CMU	7	2	27.8 ± 6.0
KKI	3	11	10.0 ± 1.5
Leuven	14	12	22.8 ± 3.6
MaxMum	15	24	26.3 ± 11.0
NYU	30	69	15.7 ± 6.6
OHSU	9	13	10.5 ± 1.6
Olin	7	11	17.2 ± 3.0
Pitt	22	21	19.0 ± 6.9
Stanford	7	9	10.1 ± 1.7
Trinity	17	24	16.9 ± 3.4
UCLA	35	29	13.1 ± 2.4
UM	9	16	16.3 ± 3.8
USM	35	22	23.8 ± 8.2
Yale	9	16	12.1 ± 2.6

**Table 2:** Cortical clusters demonstrating significant differences in 2dReHo between ASD and HC.

Cluster	Vertex number	MNI coordination	−sign⁡(t)log_10_⁡(P)
Middle frontal sulcus	52	30, −41, −18	2.86
Superior frontal sulcus	39	20, −7, −60	3.35
Precuneus gyrus	34	6, 71, −47	2.95
Middle frontal gyrus	32	33, −32, −32	3.05
Middle frontal sulcus	78	−24, −41, −32	3.27
Superior temporal sulcus	38	−45, 37, 3	4.96
Middle frontal gyrus	35	−39, −46, −20	3.28
Insular cortex	39	−38, 10, −19	−3.45
